# Rotation survival forest for right censored data

**DOI:** 10.7717/peerj.1009

**Published:** 2015-06-11

**Authors:** Lifeng Zhou, Qingsong Xu, Hong Wang

**Affiliations:** School of Mathematics and Statistics, Central South University, China

**Keywords:** Survival analysis, Censored data, Survival ensemble, Medical decision making

## Abstract

Recently, survival ensembles have found more and more applications in biological and medical research when censored time-to-event data are often confronted. In this research, we investigate the plausibility of extending a rotation forest, originally proposed for classification purpose, to survival analysis. Supported by the proper statistical analysis, we show that rotation survival forests are able to outperform the state-of-art survival ensembles on right censored data. We also provide a C-index based variable importance measure for evaluating covariates in censored survival data.

## Introduction

In biological and medical research, time-to-event data are often confronted. Survival analysis focuses on studying the relationship between covariates and the time until an event of interest occurs. The analysis has become complicated when the data are censored due to various reasons such as patients becoming uncooperative and withdrawing from a clinical trial or patients who do not experience the event (death or occurrence of a disease) when a clinical trial ends. Many parametric or semi-parametric models such as Cox-proportional hazards model and its extensions (David, 1972; Cox & Oakes, 1984) are developed to investigate such relationship in censored data. However, when the underlying assumptions are not satisfied, these models may not lead to faithful conclusions. Therefore, non-parametric models such as survival trees (LeBlanc & Crowley, 1995; Bou-Hamad, Larocque & Ben-Ameur, 2011) and neural networks (Faraggi & Simon, 1995) are evolved to relax or remove the restrictive assumptions.

Recently, ensemble-based approaches which combine one of the previous parametric and non-parametric models with state-of-the-art ensemble learning techniques are applied to create accurate and diverse base learners. Bagging, one of the most simple but ingenious ensemble techniques, was first applied with survival trees to censored data in Dannegger (2000); Benner (2002). A general bagging method for arbitrary tree growing algorithms with a conditional survival function was proposed in Hothorn et al. (2004). The popular random forest (Breiman, 2001) method was also extended to survival analysis scenario by Hothorn et al. (2006) and later by Ishwaran et al. (2008). In Hothorn et al. (2006), estimated inverse probability of censoring weights were used as sampling weights in constructing the bootstrapping samples and the final ensemble prediction is a weighted average of log-survival time predictions from all survival trees. In Ishwaran et al. (2008), Nelson-Aalen estimates of cumulative hazard functions were obtained and averaged from all nodes, and four different splitting criteria including log-rank statistics and conservation-of-event principle were provided for constructing the so-called random survival forest (RSF). The uniform consistency for RSF was further proved in Ishwaran & Kogalur (2010). Boosting techniques using different type of base learners such as regression trees (Hothorn et al., 2006; Chen et al., 2013) and smoothing splines (Lu & Li, 2008) were also studied. A Bayesian ensemble using Cox proportional hazard model, Weibull regression and accelerated failure time model for high dimensional survival data was presented in Bonato et al. (2011). All these survival ensembles have been proved to be more effective than previous monolithic models (Hothorn et al., 2004; Hothorn et al., 2006; Ishwaran et al., 2008; Ishwaran & Kogalur, 2010; Bonato et al., 2011)

In this article, we introduce rotation survival forests, a novel tree ensemble for analyzing survival data. The proposed rotation survival forest (RotSF) methodology extends the original rotation forest (RotF) approach (Rodriguez, Kuncheva & Alonso, 2006) from classification to survival analysis. In RotF, accurate and diverse classifiers are obtained through variable reconstruction by assembling information extracted from variable subsets. The randomness and diversity is introduced in two forms. First, a bootstrap sample of the training dataset is randomly generated. Second, variables are randomly divided into a number of disjoint subsets and principal component analysis (PCA) is applied in turn to each variable data subset to obtain the so-called rotation matrix. Multiplying the original dataset by the rotation matrix will lead to a new rotated dataset. These rotated datasets will be used to train tree base learners within the ensemble.

RotF is unique among all ensemble learning algorithms in that all the information in the original dataset is preserved, though in different forms of representation. Various experiments results and theoretical analysis have shown that RotF is a very competitive ensemble learning method. However, applications of RotF have focused primarily on classification problems so far. It is of a great value to generalize rotation forest to right censored survival data. Similar to RotF, our proposed rotation survival forest approach random split the *p* covariates into *k* variable subset and then some feature extraction method is executed on each variable subset. Different from RotF, coefficients of covariates which are not chosen in *k* subsets of variable are set to 0 in the rotation matrix. To further diverse the data, we employ a double bagging approach in preparing the data for rotation which in turn improves the prediction accuracy.

A byproduct of our proposed approach is that importance of variables can be calculated easily using the so-called out-of-bag (OOB) data (Breiman, 2001). The proposed importance measure based on Harrell’s C-index (Harrell, Lee & Mark, 1996) is very useful in survival analysis, as researchers usually have strong interest in determining the most significant covariates that affecting the survival probability.

In order to carry out the empirical comparisons, we establish a similar experimental framework to that in Ishwaran et al. (2008); we test on the famous public survival datasets available from public repositories. We consider an extended version of classification and regression tree (CART)(Breiman et al., 1984) as the base classifier for our rotation ensemble in that it has been the most commonly used non-parametric method in analyzing survival data (Bou-Hamad, Larocque & Ben-Ameur, 2011). To estimate prediction errors of various survival models, we use C-index as the evaluation criterion as suggested by Ishwaran et al. (2008). The results obtained in the comparisons are further validated by some proper statistical tests.

The main contributions of this paper can be summarized as follows:

• We extend the original rotation forest approach from classification to survival analysis of right censored data;
• We provide a C-index based variable importance measure for evaluating covariates in censored survival data.

## Methods

Survival analysis is the study of relationship between survival time *τ* and a set of covariates **X** = (*X*_1_, *X*_2_, …, *X_p_*). Here, *τ* is not fully observed, i.e., *τ* = *min*(*U*, *C*) is composed of both true survival time *U* and censored time *C* and an indicator variable *δ* = *I*(*U* ≤ *C*) takes 1 for true event time *U* and 0 otherwise. The major goal of survival analysis is to estimate survival experiences of different patient groups via the so-called cumulative survival function, which captures the probability that the event does not occur until a given time. In this study, we propose to model the survival function using a novel non-parametric learning ensemble called Rotation Survival Forest (RotSF).

Assume **X** to be a variable set **V** of *p* covariates and *D* be the dataset containing the training samples in a form of *n* × (*p* + 2) matrix, namely *D* = (*τ_q_*, *δ_q_*, **X**_*q*_), *q* = 1, 2, …, *n*.

In the following, we will give a high-level description of how RotSF trains a base survival learning algorithm *S_i_*:

1. Generate a bootstrap sample *D*′ of size *n* from *D*.
2. Split **V** randomly into *k* disjoint subsets *V*_*i*,*j*_(*j* = 1, …, *k*) such that each variable subset contains *M* covariates. If *p* is not divisible by *M*, there would be some variables not included in any subset and denote these remaining variables by *RV*.
3. In the *j*th iteration, generate a bootstrap sample *D*″ of size *n* from *D*′. Let *X*_*i*,*j*_ be the subset of *D*″ with variable set *V*_*i*,*j*_.
4. Run PCA on *X*_*i*,*j*_ and obtain the variable loadings matrix (rotation matrix) *M*_*i*,*j*_ for *V*_*i*,*j*_.
5. Repeat above steps 3 and 4 for all *j* = 1, 2, …, *k*, and obtain a group of *M*_*i*,*j*_*s*(*j* = 1, 2, …, *k*) for all *V*_*i*,*j*_*s*.
6. For RV covariates, set their rotations (loadings) to 0, and hence all covariates have corresponding rotation values. Then rearrange these values according to the covariates order in *V* and we will get the rearranged rotation matrix ${R}_{i}^{a}$.
7. Use the newly formed data $({\tau }_{i},{\delta }_{i},X{R}_{i}^{a})$ as the training set for the base survival tree algorithm, we will get a base survival tree learner *S_i_*.

As with most ensemble learning methods, ensemble size *L* needs to be set beforehand. Similar to RotF, a fixed value *M* = 2 is given to the size of variable subset. The base survival tree algorithm adopted here is the CART algorithm extended by LeBlanc & Crowley (1992).

The pseudo-code of the proposed RotSF algorithm is presented in Algorithm 1: 
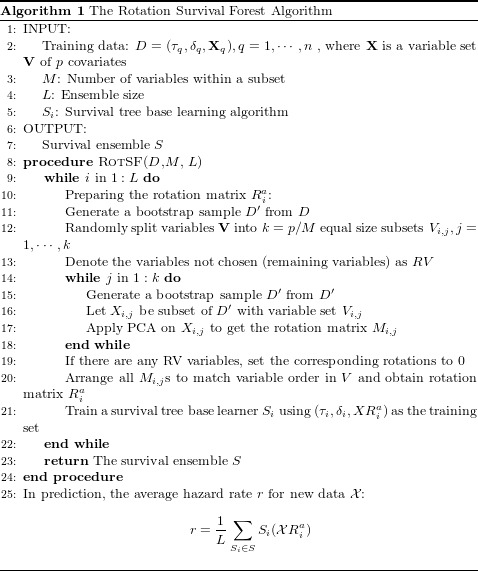


From Algorithm 1, we know that similar to random survival forest, RotSF is also a parallel algorithm and the first “while” part can be executed concurrently to save time in case of large survival data.

## Results & Discussion

In this section, we investigate the performance of the proposed RotSF model and compare with three popular survival models for censored survival data.

### Datasets

We evaluate the performance of our algorithm on three well-known survival benchmark datasets which have been extensively analyzed in the statistical literature. A brief introduction and summary of the used datasets are given below and in Table 1.

#### Primary Biliary Cirrhosis (PBC) dataset

This dataset is from the Mayo Clinic trial in primary biliary cirrhosis (PBC) of the liver conducted between 1974 and 1984. There are 418 patients in this study, 257 of whom have censored data. The currently used dataset is taken from Appendix D of Fleming & Harrington (1991).

#### Chronic Myelogenous Leukemia (CML) dataset

This dataset contains survival informatin in a randomised trial comparing three treatments for Chronic Myelogeneous Leukemia (CML). In this dataset, 507 samples, 108 of which are censored, are simulated according to structure of the data by the German CML Study Group used in Hehlmann et al. (1994).

#### Veterans’ Administration Lung Cancer (Veteran) dataset

This dataset includes survival data for 137 patients from Veteran’s Administration Lung Cancer Trial and was first made public by Kalbfleisch & Prentice (1980). There are 9 censored observations.

**Table 1:** Summary of three benchmark datasets used in the paper.

Dataset	Samples	Covariates	Censored data	Censoring rate
PBC	418	17	257	61.48%
CML	507	5	108	21.30%
Veteran	137	6	9	6.5%

### Evaluation metric & statistical tests

In medical decision making, researchers and doctors are usually concerned with the relative risks between patients with various covariates. To evaluate the accuracy of such relative risks, Harrell’s concordance index (C-index) measure was proposed in Harrell, Lee & Mark (1996). Currently, it is a widely adopted statistic in evaluating different survival models and will also be the evaluation metric in our later experiments.

To compare the performance of various survival models, the non-parametric Friedman test (Demšar, 2006) is applied. Friedman’s test statistic is based on the average ranked performance of the algorithms on each run of the datasets and can be calculated according to the following formula:

(1) \begin{eqnarray*} F T=\frac{12}{n m(m+1)}\sum _{j=1}^{m}(\sum _{i=1}^{n}r_{i}^{j})^{2}-3 n(m+1) \end{eqnarray*}

where *m* denotes the number of survival models, *n* the number of runs, and ${r}_{i}^{j}$ the rank of survival models *j* on the *i*th run. If the value of *FT* is large enough, the null hypothesis that there is no significant difference among the different survival models can be rejected and a Nemenyi post-hoc test can be adopted to find where the difference lies.

For two survival models *C*_1_ and *C*_2_, the Nemenyi statistic *z* is calculated as follows:

(2) \begin{eqnarray*} z=\frac{{R}_{j 1}-{R}_{j 2}}{\sqrt{\frac{m(m+1)}{6 n}}} \end{eqnarray*}

where *R_j_* denotes the mean rank of survival models *C_j_* on all runs of the dataset,namely, ${R}_{j}=\frac{1}{n}\sum _{i=1}^{n}r_{i}^{j}$. The performance of two survival models is significantly different if the *z* value is larger than a certain critical value (Demšar, 2006).

### Evaluating covariate importance

Covariate(variable) importance plays an importance role in the interpretation of a survival model. In this study, we introduce a new variable important measure, mean C-index decrease measure. It is based on C-index values difference on the OOB data before and after permuting the values of the variable in consideration. The variable importance for variable *i* in terms of mean C-index decrease is defined by:

(3) \begin{eqnarray*} V{I}_{i}^{c}=\frac{100}{L}\sum _{j=1}^{L}({C}_{i}-{C}_{\overline{i}}) \end{eqnarray*}

where *C_i_* and ${C}_{\overline{i}}$ denotes C-index values on the current OOB data before and after the permutation of variable *i*.

If the variable in question is not associated with the survival outcome, value permutation will have no influence on the prediction and hence no influence on the C-index. On the contrary, if outcomes and variables are indeed associated, permuting variable values results in a worse prediction power and will lead to a decrease in the C-index value. The C-index difference before and after randomly permuting the variable will reflect the importance level of the current variable. Averaging the decreased values across all survival tree base models, we will get a list of mean decrease C-index values. The higher a mean decrease C-index value is, the more important a variable would be.

### Experiment results

We conduct our experiments on a system with a Pentium Dual-Core 3.20 GHz CPU and 4 G RAM. The proposed RotSF algorithm is implemented in the R programming language. In the experiments, all training and testing sets are from a random 80% and 20% split of the benchmark datasets. Hereafter, the reported performance results are based on 1,000 random runs of RotSF and other methods.

#### Covariate importance result

**Figure 1:**
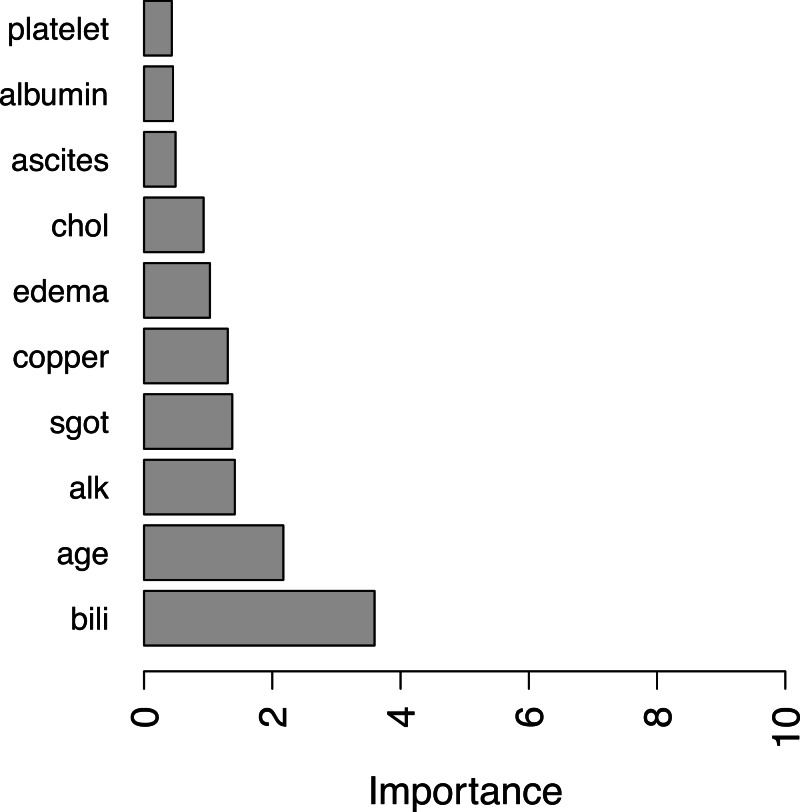
Top ten important variables by RotSF.

For illustration purpose, we calculate covariates’ importance in the PBC dataset using the above mean C-index measure. A bar plot of top 10 important covariates are shown in Fig. 1.

According the obtained result, covariates such as “bili” (serum bilirunbin), “age” are very important clinical indicators for predicting survival of patients with primary biliary cirrhosis, as permutation of these variables can cause a decrease in C-index of more than 2% on average. Compared with the results in Bou-Hamad, Larocque & Ben-Ameur (2011), one may find that there is a significant overlap in the most important covariates found by RotSF, omit- one-covariate approach and rotation survival forest. In addition, the top two covariates “bill” and “age” are the same and in the same order.

Different approaches giving similar results provides further confidence that the covariates found with C-index measure can be used in clinical decision making to evaluate the survival risks of patients.

#### The effect of double bagging

Next, we test RotSF’s performance with different bagging schemes: RotSF with double bagging (RotSF, the proposed algorithm); RotSF with single bagging (RotSFsb). For both two approaches, the same settings, i.e., *L* = 1,000 and *M* = 2 are applied. For illustration and simplicity, we only report the results with the PBC dataset in the following Table 2.

**Table 2:** RotSF’s performance with different bagging schemes.

Statistic	RotSF	RotSFsb
Min	0.7000	0.7032
1st quintile	0.8072	0.8046
Median	0.8370	0.8318
Mean	0.8347	0.8309
3rd quintile	0.8650	0.8614
Max	0.9473	0.9402

To decide whether the two bagging schemes are significantly different, a Wilcoxon signed-rank test is applied. As the *p*-value turns out to be less than 2.2e−16, we can reject the null hypothesis that these two schemes have the same predictive power. Together with the results shown in Table 2, we can come to the conclusion that double bagging outperforms single bagging under the C-index metric. This confirms our assumption that double bagging scheme can result in a more diversified training data and this does improve the algorithm’s performance.

#### Performance comparison result

Here, we compare the proposed method with three state-of-the-art survival models. The first method is Cox proportional hazard (Cox) model (David, 1972); the second method is random survival forest (RSF) and the third method is gradient boosted model (GBM) (Ridgeway, 2004). Comparisons with these models are conducted with corresponding “survival,” “randomForestSRC,” and “gbm” packages in R. For the ease of notation, survival models RotSF, RSF, Cox and GBM are denoted by A, B, C, D, respectively when necessary. In the experiments, we want all classifiers to have the same opportunities to achieve the best results, thus the default settings are adopted. For ensemble methods, i.e., RotSF, RSF and GBM, 1,000 trees are built.

**Figure 2:**
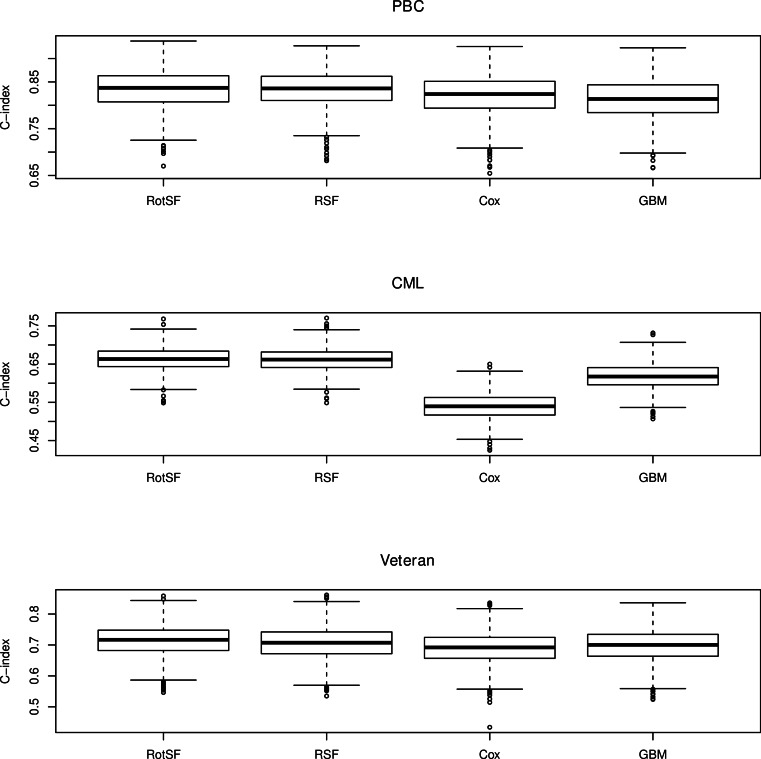
Boxplots of performance in terms of C-index.

The following Fig. 2 reports the performance of RotSF, RSF, LLR and CART algorithms in term of C-index on 1,000 runs of the experiments.

The Friedman rank sum test statistics on PBC, CML and Veteran datasets are 800.9539, 2498.443 and 403.2192, respectively and all are significant as all three *p*-values are less than 2.2e−16.

Thus, to find out which pairs of algorithms are significantly different, we compute the Nemenyi test statistics for different pairs of survival models, i.e., *z_BA_*, *z_CA_* and *z_DA_*. The post hoc Nemenyi test results are shown in the following Table 3.

**Table 3:** Friedman test and Nemenyi test results on all datasets

Dataset	Nemenyi *z_BA_*	Nemenyi *z_CA_*	Nemenyi *z_DA_*
PBC	4.849742	14.98224	26.24057
CML	2.182384	39.33487	25.82488
Veteran	4.78046	14.53191	15.39793

For *α* = 0.05, the critical value of Nemenyi’s test is 2.5742. It can be seen that all Nemenyi statistics except the *z_BA_* value on CML dataset exceed 2.5742. Thus, in terms of C-index, there exists significant differences between RotSF and the other three algorithms on PBC and Veteran datasets and also significant differences between RotSF and Cox & GBM on CML dataset. In other words, on PBC and Veteran datasets, RotSF is significantly better than RSF, Cox and GBM; on CML dataset, RotSF is also significantly better than Cox and GBM.

Tough RotSF beats RSF in 543 out 1,000 runs on CML dataset, the difference between RotSF and RSF is not significantly different at this time according to the Nemenyi statistic. However, if we repeat the experiments more times, for example, 2,000 times, the Nemenyi test statistic becomes 3.612997 and is above the critical value 2.5672. Thus, RotSF is also significantly better than RSF on CML dataset.

## Conclusion

In this study, we have developed a new ensemble learning algorithm, rotation survival forest, for survival analysis. By studying the well-known benchmark datasets, we have found that RotSF generally outperforms state-of-the-art survival models such as rotation survival forest, Cox proportional hazard and generalized boosted model in terms of C-index metric. As a non-parametric approach, RotSF does not impose parametric assumptions on hazard functions, and it extends the well-known rotation forest methodology to survival analysis.

This study also provides a mean C-index decrease measure to evaluate variable importance. The important covariates identified by RotSF agrees strongly with results reported in previous studies and may provide useful clues for clinical decision making. It is clear that other methods (ensembles and not) are available but the goal here is to illustrate some key features of the proposed method and not to provide an exhaustive comparison across methods.

The R code and the Supplemental Information are available at https://github.com/whcsu/rotsf, and we are working hard to provide an R package for the proposed RotSF algorithm as soon as possible. The proposed algorithm still has room for improvement. First, RotSF has an extra parameter which controls the variables within a subset and we just set it to 2 in our experiments. We can further test its sensitivity or use cross-validation to tune this parameter. Second, as double bagging is applied, RotSF is computationally more intensive than other ensemble methods. Fortunately, RotSF is easily parallelizable, which could help in dealing with big data. Third, same as rotation forest, PCA is chosen for feature extraction in our approach. One may try other feature extraction methods as well.

## Supplemental Information

10.7717/peerj.1009/supp-1 Supplemental Information 1 Test results on Veteran dataset-1000runs

10.7717/peerj.1009/supp-2 Supplemental Information 2 Test results on CML dataset-2000runs

10.7717/peerj.1009/supp-3 Supplemental Information 3 Test results on pbc dataset-1000run

## Additional Information and Declarations

### Data Deposition

The following information was supplied regarding the deposition of related data: Github: https://github.com/whcsu/rotsf.
